# The efficacy of mindfulness-based interventions on mental health among university students: a systematic review and meta-analysis

**DOI:** 10.3389/fpubh.2023.1259250

**Published:** 2023-11-30

**Authors:** Xinyi Zuo, Yong Tang, Yifang Chen, Zhimiao Zhou

**Affiliations:** ^1^Sociology Department, School of Government, Shenzhen University, Shenzhen, China; ^2^Institution of Policy Studies, Lingnan University, Tuen Mun, Hong Kong SAR, China; ^3^Shenzhen Senior High School, Shenzhen, China

**Keywords:** mindfulness, university students, anxiety, depression, stress, sleep quality, meta-analysis

## Abstract

**Introduction:**

We aimed to estimate the effect of mindfulness therapy on mental health.

**Methods:**

Two researchers searched 12 databases to identify relevant trials that were published from 1 January 2018 to 1 May 2023. We performed a meta-analysis to determine the effect of mindfulness therapy on depression, which was measured by the Beck Depression Inventory (BDI), Patient Health Questionnaire-9 (PHQ-9), Quick Inventory of Depressive Symptomatology (QIDS), Hamilton Depression Rating Scale (HDRS), Patient-Reported Outcomes Measurement Information System (PROMIS), Hospital Anxiety and Depression Scale (HADS), and Depression Anxiety Stress Scales (DASS); anxiety, which was measured by the Beck Anxiety Inventory (BAI), PROMIS, and DASS, Generalized Anxiety Disorder-7 (GAD-7); stress, which was measured by the Perceived Stress Scale (PSS), DASS, and GAD-7; mindfulness, which was measured by the GAD-7, Five Facet Mindfulness Questionnaire (FFMQ), Mindful Attention Awareness Scale (MAAS), Short Form-12 Mental Component Score (SF-12 MCS) and Short Form-12 Physical Component Score (SF-12 PCS); and sleep quality, which was measured by the Pittsburgh Sleep Quality Index (PSQI). After screening studies based on the inclusion and exclusion criteria, 11 randomized controlled trials (RCTs) involving 1,824 participants were ultimately included.

**Results:**

All these studies demonstrated positive effects of mindfulness therapy on depression (SMD = −0.33, 95% CI: [−0.44, −0.22], *p* < 0.00001, I2 = 29%), anxiety (SMD = −0.35, 95% CI: [−0.46, −0.25], *p* < 0.00001, I2 = 40%), stress (SMD = −0.39, 95% CI: [−0.48, −0.29], *p* < 0.00001, I2 = 69%) and sleep quality scores (SMD = −0.81, 95% CI: [−1.54, −0.09], *p* = 0.03, I2 = 0%). However, there was no significant difference in mindfulness (SMD = −0.12, 95% CI: [−0.36, −0.12], *p* = 0.34, I2 = 34%) between the mindfulness therapy group and the control group.

**Discussion:**

In future studies, it is necessary to consider the investigation on whether the strategies of improving the mindfulness therapy in adherence and fidelity can work on the improvement of the outcomes in mental health.

**Systematic Review Registration:**

https://www.crd.york.ac.uk/PROSPERO/, https://www.crd.york.ac.uk/PROSPERO/, identifier [CRD42023469301].

## 1 Introduction

Anxiety and depression are the most common mental disorders. Due to their negative impact on work capability and performance, mental disorders have received widespread attention. When young people go to college, they experience a variety of stressors, such as leaving home and becoming independent, assuming new responsibilities, and embracing new academic challenges (1). In addition to the effects of academic and social lives and personal habits, this sudden independence brings numerous choices to students. As a result, many college students report increasing levels of stress and an inability to cope with their stressors, thus leading to an overwhelming feeling (2). Frequently, college students make sacrifices in some major aspects of life, and sleep quality is thus often neglected. To this end, early psychological interventions may contribute to the prevention of mental disorders (3). Moreover, the prevalence of mental health disorders varies widely among university students; in some countries, the prevalence of depressive symptoms reaches up to 50% (4). Therefore, it is necessary to develop a simple, economical, feasible and effective intervention for addressing mental health issues among university students.

To date, researchers have developed many mental health interventions. Among them, mindfulness therapy is recommended as a nonpharmacological treatment method because it has very few side effects (5, 6). According to a recent meta-analysis, potential advantages of mindfulness therapy include lower levels of depression (7), anxiety (8) and stress (9) as well as improvements in sleep quality (10) among both university students with clinical symptoms and healthy individuals. Mindfulness therapy originates from a tradition of Buddhism in the east, which has a history of more than 2,500 years. Mindfulness mainly entails (1) paying attention to the current moment, (2) being nonjudgmental, and (3) being intentional (11). Moreover, mindfulness is defined as an individual’s ability to account for the details of currently occurring events (12). According to the use context, mindfulness has the following definitions: first, a method of concentrating the mind; second, a meditation technique; third, a skill; and fourth, a specific method of treatment (13). The essential components of mindfulness interventions include awareness cultivation, experience enhancement, responsiveness fostering, and tolerance increase (14). As a kind of effective intervention, mindfulness training (or interventions based on mindfulness) has gained popularity among scientists circle and within the general public due to its role in promoting well-being and health. With the adaptation of oriental Buddhist practices and techniques, mindfulness training was first introduced to Western culture in the 1970s. It was designed to alleviate stress as well as reduce psychological distress through numerous secular and contemplative practices (15). Subsequently, many mindfulness training with varying contents have been developed; they share the same objective of paying attention to and fostering awareness of experiences at the current moment with a nonjudgmental, accepting attitude (16). There are numerous types of nonreligious psychological interventions centered around mindfulness, including mindfulness-based cognitive therapy (MBCT) (17, 18), mindfulness-based stress reduction (MBSR) (11, 19) and brief mindfulness meditation training (20, 21). Many interventions based on mindfulness incorporate mindfulness training into an all-around treatment program as an essential part, such as acceptance and commitment therapy (ACT) (22–24) and dialectical behavior therapy (DBT) (25, 26). Additionally, many interventions include mind–body training (27). The operating mechanism is to concentrate on the current moment with a nonjudgmental attitude while preventing oneself from being absent-minded, thereby triggering the reperception experience and achieving emotional regulation. In this way, negative emotions can be effectively reduced (28). Currently, mindfulness training is well established in cognitive–behavioral therapy (CBT), which is also most prominently used in manualized and structured group settings, such as groups practicing stress reduction based on mindfulness (11) or cognitive therapy based on mindfulness (29). Among these interventions, students frequently practice mindfulness in groups and through daily assignments. However, such widely applied mindfulness training is still in need of careful scrutiny.

On one hand, mindfulness therapy has been viewed as a potential clinical intervention strategy for treating mental health (30). In addition, due to its significant effects, mindfulness has become popular among healthy subjects (31). Many studies have reported that college students may benefit from mindfulness interventions (32–35).

However, conflicting results were recently reported in two meta-analyses. Vøllestad et al. (36) assessed the effects of mindfulness-based interventions (MBIs) on individuals suffering anxiety disorders; they reported significant reductions in both anxiety (g = −0.83) and depression symptoms (g = −0.72). On the other hand (37), assessed the effects of MBIs on individuals with anxiety and depression disorders; they failed to find an obvious effect of MBIs on anxiety disorders (*p* = 0.09). Thus, clinical data on the effect of mindfulness therapy on mental health remain controversial. Hence, it is essential to assess both the effect of mindfulness therapy and the factors that contribute to the efficacy of MBIs, such as the treatment duration, group vs. individual formats, and the target groups. Due to the inconsistencies in these factors among the abovementioned meta-analyses, it is difficult to determine the underlying reasons for the different findings. Hence, we conducted a systematic review and meta-analysis of randomized controlled trials (RCTs) to evaluate the effect of mindfulness therapy on mental health and further provide a reference for clinical practice.

## 2 Methods

This systematic review was performed in accordance with the Preferred Reporting Items for Systematic Reviews and Meta-Analyses (PRISMA) guidelines (Multimedia Appendix 1 Checklist 2020) (38). We registered the study in PROSPERO (CRD42023469301).

### 2.1 Selection criteria

In this study, the eligibility criteria were made in accordance with the PICOS principles. (1) P: The subjects had at least one indicator indicating emotional problems, and their age was 16 years or older. (2) I: MBIs (mindfulness-based interventions) were implemented among university students in the experimental group (e.g., ACT, MBSR, DBT, MBCT, mindfulness walking in nature, mindfulness meditation, mind–body training) without restrictions on the time of intervention. (3) C: The control group received a different intervention (e.g., routine health care, wait-list, general conversation). (4) O: The outcomes included depression, anxiety, mindfulness or sleep quality in college students. (5) S: The type of study was RCTs.

The exclusion criteria were as follows: (1) reviews, non-RCTs, articles for which the full text was unavailable, or case reports; (2) duplicate publications or animal experiments; and (3) incomplete or unavailable data.

Moreover, the PICOS principles were used to identify eligible studies (Table 1). All included studies were published in Chinese or English. Studies wherein the results are interpreted from the perspective of college students were deemed eligible.

**Table 1:** PICOS-based eligibility criteria (participation, intervention, comparison, outcomes, and study design).

PICOS	Criteria
Participation	College students
Intervention	MBI
Comparison	MBI group and Wait-list group
Outcome	Anxiety, Depression, Stress, Mindfulness or Sleep quality
Study design	Randomized controlled trial

### 2.2 Search strategy

The following database were searched to identify literature: PubMed, the UWE Library database, MEDLINE, the Cochrane Library, Embase, PsycINFO, Scopus, SinoMed, China National Knowledge Infrastructure (CNKI), Wanfang Data, Wanfang MED ONLINE, Yiigle and Web of Science.

The search terms used in the different databases were slightly different. Keywords such as “quality or Mood or Stress,” and “a pilot study or Randomized Controlled Trial or RCT,” and “Mindfulness-Based or Mindfulness or Web-Based or Mobile,” as well as “University student or College student or Young people” were used to search for articles published from 1 January 2018 to 1 May 2023. The “snowball” method was adopted to trace the references of the included literature. Additionally, the references of the included studies were manually searched to identify eligible articles. Unpublished academic literature was not considered to be eligible. The search strategy for the PubMed databases is shown in Box 1.

**Box 1 PubMed retrieval strategy.**

#1 “Mindfulness-Based or Mindfulness”(All Fields) OR “Web-Based or Mobile”(All Fields)

#2 “Mood”(Title/Abstract) OR “Depression”(Title/Abstract) OR “Anxiety”(Title/Abstract) OR

“Stress”(Title/Abstract) OR “Sleep quality”(Title/Abstract)

#3 #1 AND #2

#4 “University student”(Title/Abstract) OR “College student”(Title/Abstract) OR “Young people”(Title/Abstract)

#5 “A pilot study”(Title/Abstract) OR “Randomized Controlled Trial”(Title/Abstract) OR “RCT”(Title/Abstract)

#6 #3 AND #4 AND #5

**Figure 1:**
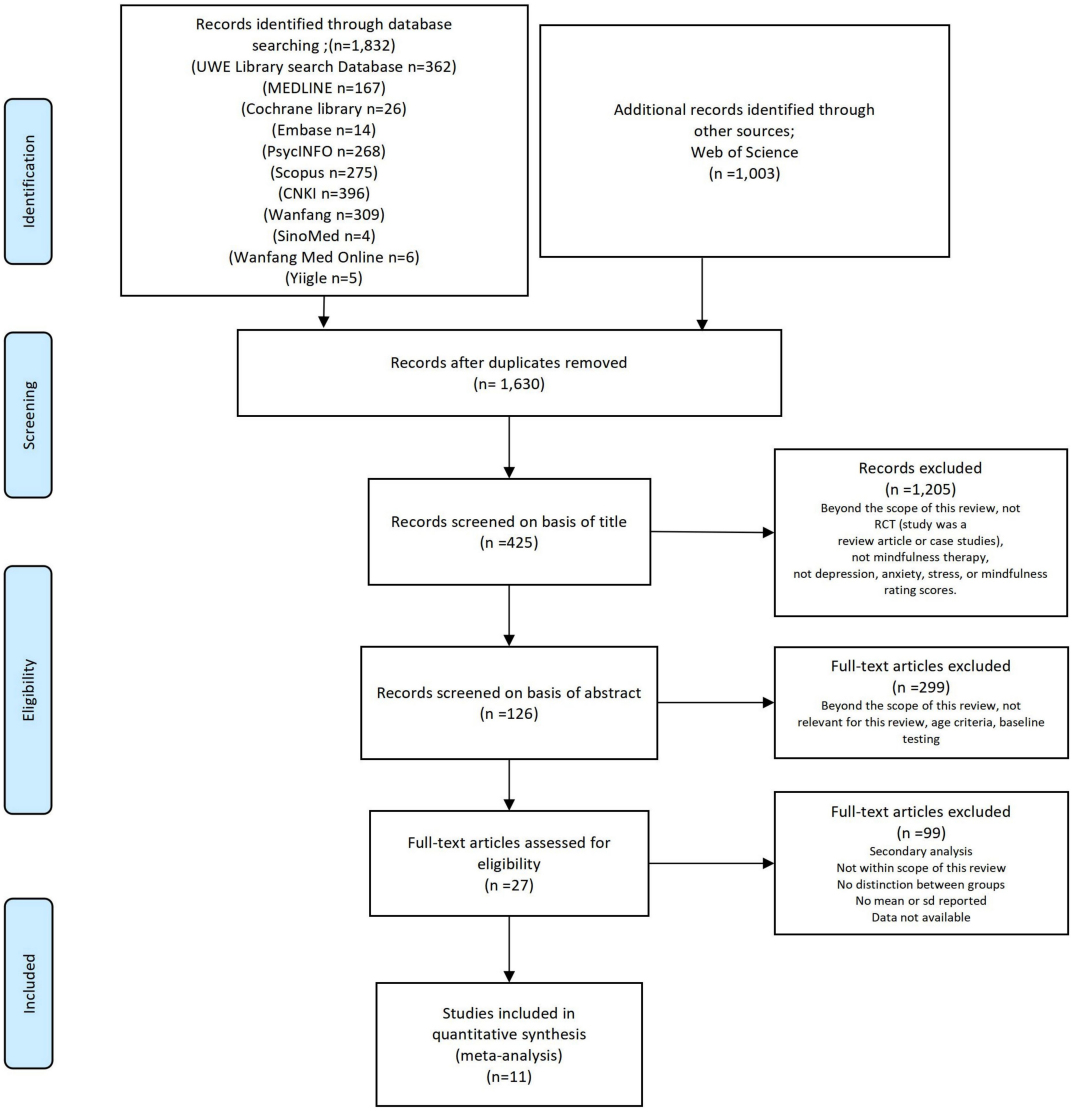
Study selection flowchart (PRISMA, 2020).

The search strategies are demonstrated in Figure 1. Four researchers (ZXY, TY, CYF and ZZM) screened all the literature for inclusion. After removing duplicates, all studies were initially screened based on titles and abstracts. Then, the researchers carefully read the full texts of the remaining articles in accordance with the inclusion and exclusion criteria. Finally, the data were extracted from the included literature. For the identification of further related publications, we also retrieved the gray literature (opengrey.eu).

### 2.3 Data extraction

After the removal of duplicate studies (EndNote X9). Four reviewers (ZXY, TY, CYF and ZZM) independently screened the titles and abstracts of the studies. Then, the full texts of the remaining studies were evaluated in accordance with the inclusion and exclusion criteria.

The following data were extracted from the included studies: (1) the information of the literature included (name of author, region, publication year, and publication type); (2) characteristics of the subjects (sample size, participant age, participant details, participant health conditions); (3) interventions (setting, intervention type, control conditions, intervention dose, study design, intervention provider); (4) information on quality of the study (Jadad score); and (5) main outcomes.

### 2.4 Assessment risk of Bias and quality

Using the Cochrane Collaboration Risk of Bias tool (39). Four researchers (ZXY, TY, CYF and ZZM) independently assessed the risk of bias. Additionally, the quality of the literature was assessed using the Jadad scale (40). The Risk of Bias tool assesses seven domains: 1. random sequence generation; 2. concealment of allocation; 3. subjects and experimenter blinding; 4. outcome assessor blinding; 5. resulting data integration; 6. selective reports; 7. other risk of bias. Each study was categorized as having an unclear risk of bias, low risk of bias or high risk of bias. We also performed the Begg and Egger tests to evaluate the degree of publication bias (41).

### 2.5 Statistical analysis

Data analysis was performed using Review Manager 5.3 software and stata15.1. Forest maps were constructed to intuitively illustrate the results. In the included literature, the outcomes were measured as continuous variables, and the same indicator was assessed by different tools. Such indicators were represented as standard mean differences (SMDs). α = 0.05 was used to indicate significance. The heterogeneity was assessed by I^2^ statistics, which were categorized as low (<50%), moderate (50–75%) or high (>75%) (42). In the case of high heterogeneity, sensitivity analysis was performed using the leave one out method to identify the sources of heterogeneity. In the analysis, numerous weeks were compared as subpoints to check the results. Begg’s test (43) and Egger’s test (44) were employed to check the possibility of publication bias. *p* < 0.05 indicated significant results. When at least 10 studies were included in a meta-analysis, a funnel plot (45, 46) was used to assess the publication bias. In this study, the standard mean difference (SMD) and 95% CI were examined (47). According to the overall effect, when p < 0.05, MBIs had statistically significant effects.

### 2.6 Subgroup analyses

Subgroup analyses were conducted based on country of intervention (outside Europe or not) and length of intervention (weeks).

## 3 Results

### 3.1 Selection of studies

Figure 1 shows the screening process, and the screening results are shown in Figure 1. After searching 12 databases, 2,835 relevant records were identified. For the purpose of duplicate removal, we imported all studies into EndNote X8 (Bld, 10,063) (48). After the removal of 1,528 duplicates and the elimination of 1,296 articles by a strict screening process, 11 trials (49–59) involving 1,824 participants were ultimately included. Studies were excluded due not reporting the sd values (60), being a review article (61), and not being within the scope of this metaanalysis (the study used a mindfulness intervention (MIND), in contrast to the intervention plus support from nonspecialist peer counsellors (MIND+) (62). All the included studies reported a positive impact of mindfulness on mental health, and the primary outcomes of interest were a reduction in depression, assessed by the Beck Depression Inventory (BDI), Hamilton Depression Rating Scale (HDRS), Patient Health Questionnaire-9 (PHQ-9), Quick Inventory of Depressive Symptomatology (QIDS), Hospital Anxiety and Depression Scale (HADS), Depression Anxiety Stress Scale (DASS), and Patient-Reported Outcomes Measurement Information System (PROMIS); a reduction in anxiety, assessed by the Beck Anxiety Inventory (BAI), PROMIS, and DASS, and the Generalized Anxiety Disorder-7 (GAD-7); a reduction in stress, assessed by the Perceived Stress Scale (PSS), DASS, and GAD-7; and improved sleep quality, measured by by the Pittsburgh Sleep Quality Index (PSQI). However, there was no significant difference in mindfulness scores, mindfulness, measured by the GAD-7, Five Facet Mindfulness Questionnaire (FFMQ), Mindful Attention Awareness Scale (MAAS), Short Form-12 Mental Component Score (SF-12 MCS) and Short Form-12 Physical Component Score (SF-12 PCS).

### 3.2 Study characteristics

Table 2 shows the overall characteristics of the included studies. All eleven studies were published before 2023 (Table 2). The sample sizes ranged from 52 to 386, and 1,824 college students above 16 years old were enrolled in the included studies, including 846 participants in the experimental group and 849 participants in the control group. All participants were university students with mood disorders, but they had not been diagnosed with a psychiatric disorder. All the interventions were based on mindfulness, and their durations ranged from 15 days to 2 months. The interventions took from 0.5 to 1.5 h each week. Both group training and individual training methods were used (49–59). All studies (49–59) were subdivided into training methods (mindfulness-based intervention) such as MBSR, ACT, MBCT, DBT, mindfulness walking in nature, mindfulness meditation, and mind–body training.

**Table 2:** Characteristics of the included studies.

References	Country	Publication type	Age (years)		Sample size	Population group	Health condition	Type of intervention	Control conditions
MG	CG	(MG/CG)
1. Shufang Sun et al. (49)	China	Journal article	18 years or older	18 years or older	*n* = 114 (MG: 57, CG: 57)	College students	Experiencing elevated psychological distress, such that their depression or anxiety symptoms were at or above the mild cutoff on the PHQ-9 and GAD-7.	Mindfulness-based mobile health intervention	Remote social support (as usual)
2. Paul Ritvo et al. (50)	Canada	Journal article	18 years or older	18 years or older	*n* = 154 (MG: 76, CG: 78)	Undergraduate students	Beck Depression Inventory-2 (BDI-II) score indicating at least mild severity, with no upper limit (BDI-II score ≥ 14)	CBT-M	Waitlist control
3. Küchler, A.-M et al. (51)	Germany	Journal article	18 years or older	18 years or older	*n* = 386 (MG: 130, CG: 127)	Undergraduate students	Moderate to low mindfulness (FMI ≤37)	StudiCare-M	Waitlist control
4. Christo EI Morr et al. (52)	Canada	Journal article	18 years or older	18 years or older	*n* = 159 (MG: 79, CG: 80)	Undergraduate students	Depression, anxiety, and stress but no indicated substance abuse or episodes of psychotic behaviors during the month prior to the trial.	Web-based Mindfulness Virtual Community intervention	Waitlist control
5. Jingni Ma et al. (53)	UK	Journal article	16 years or older	16 years or older	*n* = 101 (MG: 52, CG: 49)	Undergraduate students	Self-identified as experiencing some level of sleep difficulties	Mindful walking in nature	Waitlist control
6. Simonsson et al. (54)	UK	Journal article	Aged 18–24 years	Aged 18–24 years	*n* = 17 (MG: 88, CG: 89)	University students	Anxiety and depression	Online mindfulness intervention	Waitlist control
7. Ahmad et al. (55)	Canada	Journal article	18 years or older	18 years or older	*n* = 78 (MG: 39, CG: 39)	Undergraduate students	Depression, anxiety, and stress symptoms.	Web-based full Mindfulness Virtual Community intervention	Waitlist control
8. Huberty et al. (56)	USA	Journal article	18 years or older	18 years or older	*n* = 88 (MG: 41, CG: 47)	Undergraduate students	Scored ≥14 points on the PSS	Mindfulness meditation mobile app “Calm” intervention	Waitlist control
9. Brian J. Hall et al. (57)	Macao (Mainland China)	Journal article	18 years old and above	18 years old and above	*n* = 52 (MG: 27, CG: 25)	Undergraduate students	Mental disorders and sleep dysfunction	A low-intensity health-enhanced mindfulness intervention	Waitlist control
10. Anna F. Dawson et al. (58)	Germany	Journal article	18 years old and above	18 years old and above	*n* = 149 (MG: 74, CG: 75)	Undergraduate students	Have a moderate to low level of mindfulness according to a cutoff of <37 on the Freiburg Mindfulness Inventory	The StudiCare project offers internet and mobile-based interventions (IMI)	Waitlist control
11. Krifa et al. (59)	Republic of Tunisia	Journal article	Aged 18–30 years	Aged 18–30 years	*n* = 366 (MG: 183, CG: 183)	Healthcare students	Internet-based positive psychology program	Internet-based positive psychology intervention	Waitlist control

References	Intervention		Setting	Study design	Intervention dose	Intervention provider	Main outcome	Jadad score	MG	CG
1. Shufang Sun et al. (49)	MT	WI	Clinical	RCT	Weekly one-hour meetings (4 weeks)	Licensed psychologist, MBI teacher, Zoom	Anxiety:a-7; Depression: b-9; Mindfulness scores: c;	6
2. Paul Ritvo et al. (50)	MT	WI	Clinical	RCT	12 sessions of 20-min video conferences (8 weeks)	Moderator psychologist, online	Depression: d/e/f-24; Anxiety: g; Stress: h;	4
3. Küchler, A.-M et al. (51)	MT	WI	Clinical	RCT	Weekly sessions for 45–60 min each session, 8 weeks for the core intervention and 6 months for the entire intervention (8 weeks)	E-coaches (psychologists trained and supervised by the authors)online	Depression:b-9; Anxiety:a-7; Stress:i-4,; Mindfulness scores: j;	4
4. Christo EI Morr et al. (52)	MT	WI	Clinical	RCT	30 min, via a smartphone or laptop (2 months)	Online	Depression: b-9; Anxiety: g; Stress: h-5F,i;	6
5. Jingni Ma et al. (53)	MT	WI	Clinical	RCT	Walk in the forest or on the road for 30–35 min of daytime mindfulness (15 days)	Previously published guidance	Stress: i; Sleep quality:l;	4
6. Simonsson et al. (54)	MT	WI	Clinical	RCT	Weekly classes via Zoom, 90 min each (8 weeks)	Mindfulness teacher, Zoom	Depression & Anxiety: n;	5
7. Ahmad et al. (55)	MT	WI	Clinical	RCT	Informed by cognitive behavioral therapy (CBT) constructs (8 weeks)	A mental health professional; online	Depression:b-9; Stress:h,i-10;	6
8. Huberty et al. (56)	MT	WI	Clinical	RCT	At least 10 min per day (8 weeks)	Online	Stress: h, i;	6
9. Brian J. Hall et al. (57)	MT	WI	Clinical	RCT	Two group training sessions (each last for 1.5 h) and 7 weeks of home-based audio-guided mindfulness practice (7 weeks)	Master’s degree in counseling psychology, WeChat messages through a smartphone or tablet computer	Depression & Anxiety & Stress: k-21; Sleep quality: l;	5
10. Anna F. Dawson et al. (58)	MT	WI	Clinical	RCT	(6 weeks)	online	Depression: b-9; Anxiety: m-7; Stress: o-20; Mindfulness scores: p, q;	3
11. Krifa et al. (59)	MT	WI	Clinical	RCT	88 sessions (8 weeks), approximately 45 min each	Virtual instructors, online (videos)	Depression and Anxiety and Stress:k-21	4

Group: MG, mindfulness group; CG, control group; Intervention: MT, mindfulness therapy; WI, without intervention; RC, routine care; Study design: RCT, randomized controlled trial. Main outcome: a: GAD, generalized anxiety disorder screener; b: PHQ, patient health questionnaire for depression measurement; c: MAAS, the mindful attention awareness scale; d: BDI, beck depression inventory; e: QIDS, quick inventory of depressive symptomatology; f: HDRS, the Hamilton depression rating scale; g: BAI, beck anxiety inventory; h: FFMQ-5F, perceived stress scale; i: PSS, the perceived stress scale; j: WHO, World Health Organization Well-Being Index; k: DASS, depression, anxiety, and stress scales; l: PSQI, the Pittsburgh sleep quality index; m: GAD, generalized anxiety disorder; n: PROMIS, patient-reported outcome measurement information system (anxiety and depression scales); o: PSQ, perceived stress questionnaire; p: SF-12 PCS, short form health survey-12 physical component scale; q: SF-12 MCS, SF12 mental component scale.

### 3.3 Risk of Bias and quality assessment

**Figure 2:**
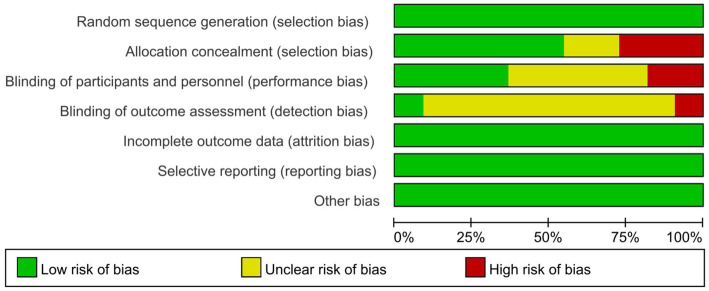
Risk of bias graph.

**Figure 3:**
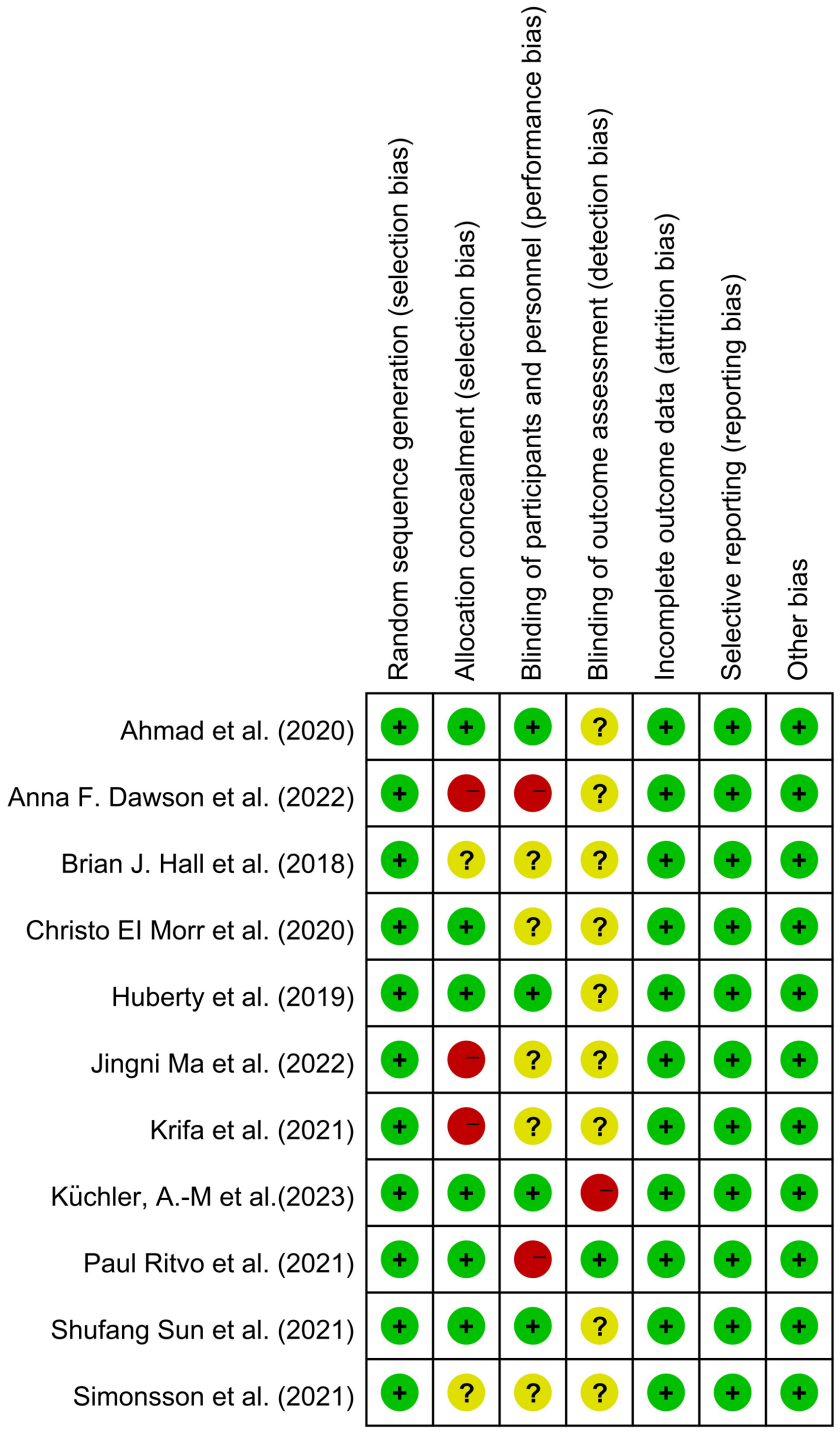
Risk of bias summary of included studies.

Figure 2 shows the risk of bias assessment. All 11 articles (49–59) described the randomization methods in detail. The blinding method was detailed in four studies, and all were single-blinded trials (49, 51, 55, 56). Two articles reported the dropout rate (49, 57). The average Jadad score across all included studies was 4.9, indicating fair to mild quality (Figure 3).

### 3.4 Meta-analyses

#### 3.4.1 Depression scores

**Figure 4:**
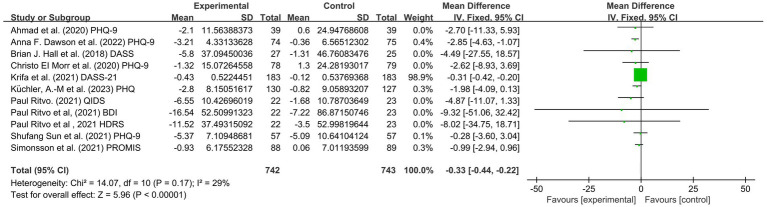
Forest plot for depression.

Nine included studies (49–52, 54, 55, 57–59) involving 1,635 university students (753 from the experimental group and 753 from the control group) assessed the effects of MBIs on college students’ depression scores based on the PHQ, DASS, QIDS, BDI, HDRS and PROMIS. Because different evaluation tools were used, the SMD was employed as the pooled effect size measure. There was a low degree of heterogeneity among studies (*p* = 0.17, I^2^ = 29%). The MBI group had lower depression scores than the control group (SMD = 0.33, 95% CI: 0.44 to −0.22, *p* < 0.00001). The results indicated that the MBI significantly alleviated the depressive symptoms scores of university students (Figure 4).

#### 3.4.2 Anxiety indicators

**Figure 5:**
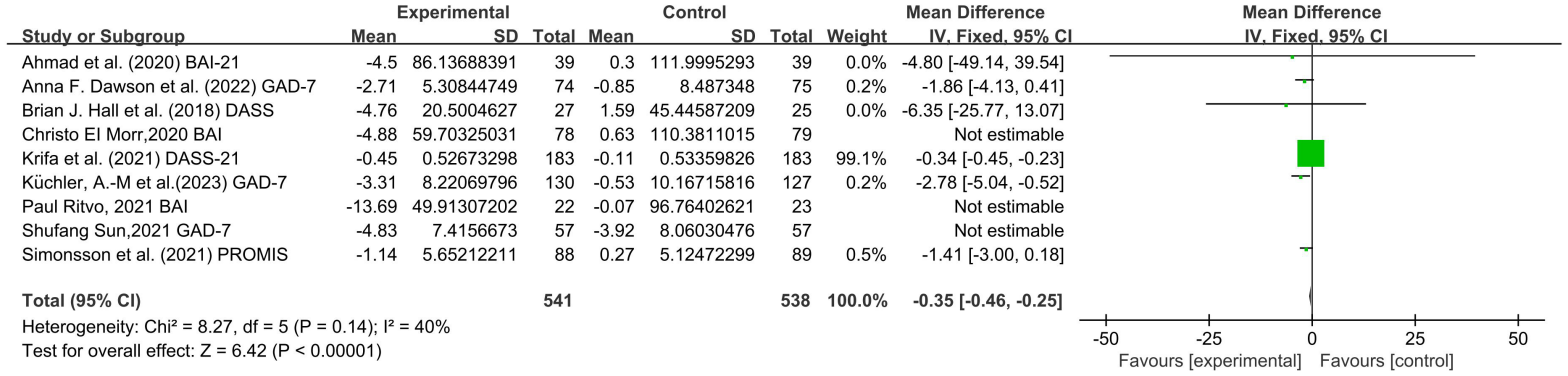
Forest plot for anxiety.

Nine articles involving 1,635 subjects, including 753 students in the experimental group and 753 students in the control group, assessed anxiety. There was a medium level of heterogeneity (I^2^ = 40%, *p* = 0.14). The mindfulness group had significantly lower anxiety scores than the control group [SMD = −0.35, 95% CI (−0.46, −0.25), *p* < 0.00001], indicating that the mindfulness intervention had a significant effect on the reduction of anxiety among college students (see Figure 5).

#### 3.4.3 Stress indicators

**Figure 6:**
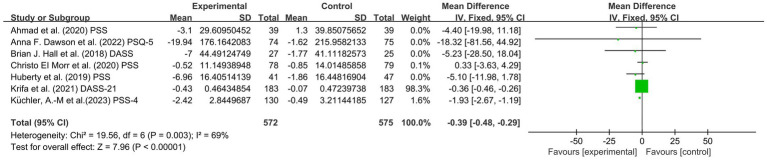
Forest plot for stress.

Seven articles involving 1,278 subjects, including 573 students in the experimental group and 576 students in the control group, assessed stress. There was a high degree of heterogeneity (I^2^ = 69%, *p* = 0.003). The mindfulness group had significantly lower stress score than the control group [SMD = −0.39, 95% CI (−0.48, −0.29), *p* < 0.00001]. This finding indicated that there was a significant effect of mindfulness on the reduction of stress levels among college students (see Figure 6).

#### 3.4.4 Mindfulness score indicators

**Figure 7:**
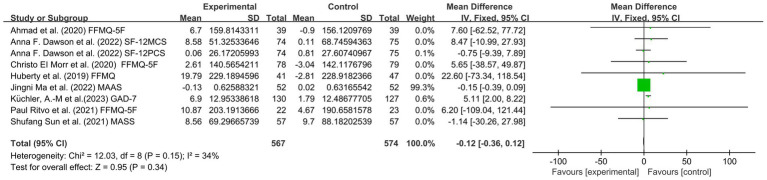
Forest plot for mindfulness.

Eight articles involving 1,229 subjects, including 548 students in the experimental group and 552 students in the control group, assessed mindfulness scores. There was a low level of heterogeneity (I^2^ = 34%, *p* = 0.15). The mindfulness group had significantly lower mindfulness scores than the control group [SMD = −0.12, 95% CI (−0.36, −0.12), *p* = 0.34]. This finding indicated that there was no significant effect of MBIs on the reduction of mindfulness scores among college students (see Figure 7).

#### 3.4.5 Sleep quality indicators

**Figure 8:**
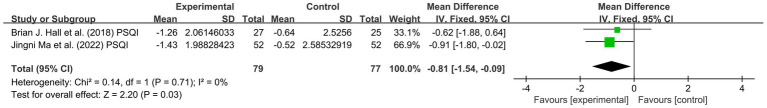
Forest plot for sleep quality.

Two articles involving 153 subjects, including 79 students in the experimental group and 74 students in the control group, assessed sleep quality. There was no heterogeneity (I^2^ = 0%, *p* = 0.71). The mindfulness group had significantly higher sleep quality scores than the control group [SMD = −0.81, 95% CI (−1.54, −0.09), *p* = 0.03]. This finding indicated that the mindfulness intervention training had improved the improvement of sleep quality among college students (see Figure 8).

In conclusion, the included studies reported scores on the PHQ-9, BDI, QIDS, HDRS, HADS, PROMIS, DASS, GAD-7, BAI, PROMIS, PSS, DASS, GAD, MAAS, FFMQ scale, SF-12 PCS and SF-12 MCS. A total of five outcomes were evaluated: depression, anxiety, stress, mindfulness scores and sleep quality. The mindfulness group (MG) had significantly lower total scores for depression symptoms than the control group (SMD = −0.33, 95% CI: [−0.44, −0.22], *p* < 0.00001, I^2^ = 29%; Figure 4). In addition, a reduction in stress was observed based on the PSS, DASS, and GAD-7. The MG had reduced anxiety symptoms (SMD = −0.35, 95% CI: [−0.46, −0.25], *p* < 0.00001, I^2^ = 40%; Figure 5) and stress symptoms (SMD = −0.39, 95% CI: [−0.48, −0.29], *p* < 0.00001, I^2^ = 69%; Figure 6) compared to the CG. Moreover, an increased in sleep quality (SMD=−0.81, 95% CI: [−1.54, −0.09], *p* = 0.03, I^2^ =0%). However, there was no statistically significant difference in mindfulness symptoms (SMD = −0.12, 95% CI: [−0.36, −0.12], *p* = 0.34, I^2^ = 34%; Figure 7) or between the MG and CG (Figure 8).

### 3.5 Subgroup analyses

Subgroup analyses of the anxiety, depression and sleep quality scores were performed based on the continent and duration of the intervention (weeks).

#### 3.5.1 Continent

**Figure 9:**
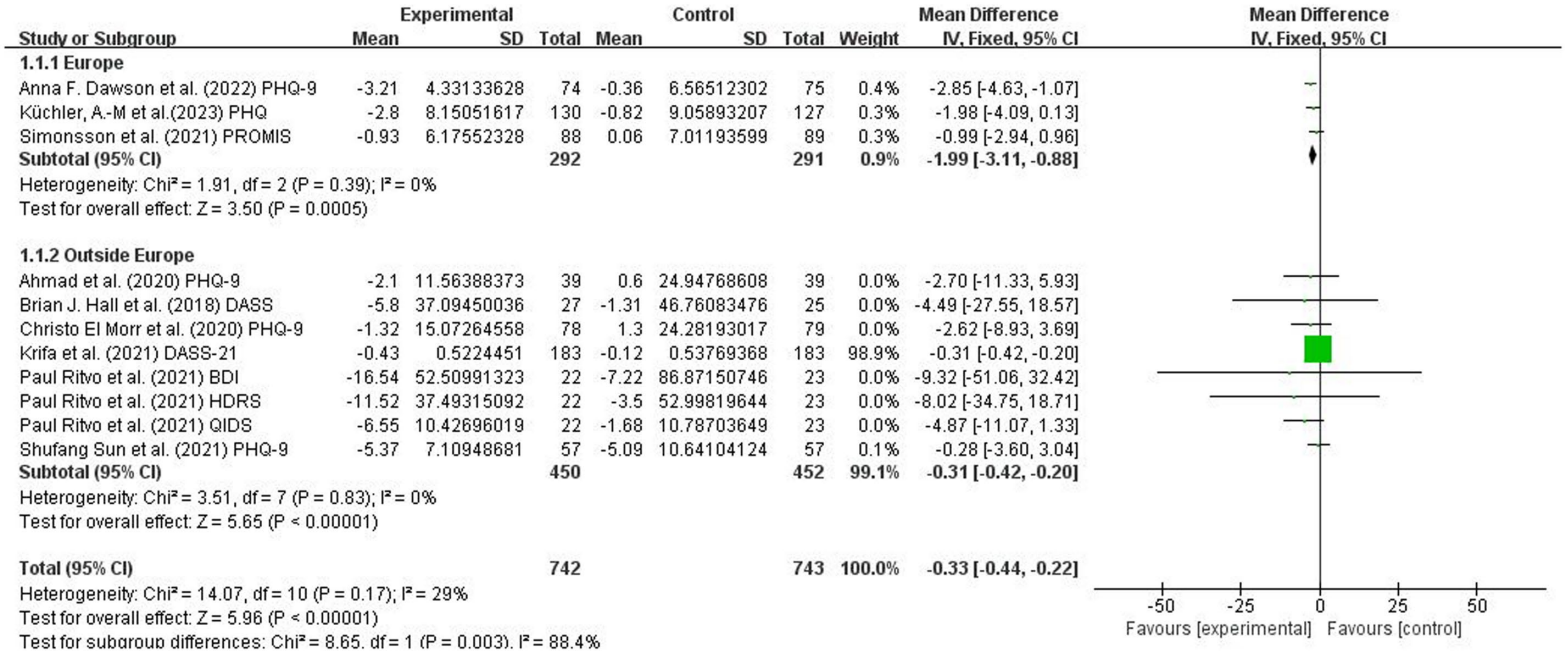
Forest graph showing subgroup analysis for depression.

For depression, significant differences were found in the SMD of the two subgroups: Europe (51, 54, 58) (*p* = 0.0005) and outside Europe (49, 50, 52, 55, 57, 59) (*p* < 0.00001). MBIs showed a significant effect on the outcome in the outside Europe group (SMD = −0.31, 95% CI: −0.42 to −0.20, *p* < 0.00001; see Figure 9). For anxiety, significant differences were found in the SMD of the two subgroups: Europe (51, 54, 58) (*p* = 0.001) and outside Europe (49, 52, 55, 57) (*p* < 0.00001). MBIs showed a significant effect on the outcome in both the Europe (SMD = −1.86, 95% CI: −2.99 to −0.73, *p* = 0.001) and outside Europe groups (SMD = −0.34, 95% CI: −0.45 to −0.23, *p* < 0.00001). For stress, there were significant differences in the SMD between the two subgroups: Europe (51, 58) (*p* < 0.00001) and outside Europe (52, 55–57, 59) (*p* < 0.00001). MBIs showed a significant effect on the outcome in both the Europe (SMD = −1.93, 95% CI: −2.67 to −1.19, (*p* < 0.00001) and outside Europe groups (SMD = −0.36, 95% CI: −0.46 to −0.26, (*p* < 0.00001). However, for mindfulness scores, no significant differences in SMD were found in the two subgroups: Europe (51, 53, 58) (*p* = 0.34) and outside Europe (49, 50, 52, 55, 56) (*p* = 0.80). MBIs did not show significant intervention effects in either the Europe (SMD = −0.12, 95% CI: −0.36 to 0.12, *p* = 0.34) or outside Europe groups (SMD = 2.89, 95% CI: −19.04 to 24.83, *p* = 0.80). In terms of sleep quality, there were no significant differences in the SMD of the two subgroups: Europe (53) (*p* = 0.04) and outside Europe (57) (*p* = 0.33). MBIs did not show significant intervention effects in either the Europe (SMD = −0.91, 95% CI: −1.80 to 0.02, *p* = 0.04) or outside Europe groups (SMD =0.62, 95% CI: −1.88 to 0.64, *p* = 0.33).

#### 3.5.2 Intervention duration (weeks)

Regarding depression, 6 out of the 9 studies showed a pooled effect (51, 52, 54, 55, 59) with a duration of intervention of ≥8 weeks [SMD = −0.32, 95% CI: (−0.43 to −0.21), *p* < 0.00001]. For the remaining three studies (49, 57, 58), the pooled effect within the intervention period was [SMD = −2.29, 95% CI (−3.85, −0.72), *p* = 0.04]. Compared with the control group, the effects of mindfulness therapy on depression during the two periods of intervention were significantly different, and a significant difference between the two groups was found (*p* < 0.00001). It was found that an intervention period of 8 weeks or more had a significant effect on reducing college students’ levels of depression. For anxiety, no significant differences were shown in the SMD of the two subgroups with a period of more than 8 weeks (51, 52, 54, 55, 59) (*p* < 0.00001) or <8 weeks (49, 57, 58) (*p* = 0.09). Among the 9 studies of stress indicators, 5 of them had a combined effect (51, 52, 55, 56, 59) in an intervention cycle ≥8 weeks [SMD = −0.39, 95% CI: (−0.48 to −0.29), *p* < 0.00001]. For two studies (57, 58), the combined effect within the intervention period was [SMD = −6.79, 95% CI (−28.63, −15.05), *p* = 0.54]. Within comparison with the control group, mindfulness therapy’s effects in the two intervention periods on stress were significantly different, and a significant difference was found between the groups (*p* < 0.00001). Intervention lasting ≥8 weeks can significantly reduce college students’ stress levels. Regarding mindfulness scores, no significant differences in SMD were found between the interventions lasting ≥8 weeks (50–52, 55, 56) (*p* = 001) and those lasting <8 weeks (49, 53, 58) (*p* = 0.23). MBIs were not found to have a significantly different effects based on whether the intervention lasted ≥8 weeks (SMD = −5.14, 95% CI: 2.04 to 8.23, *p* = 0.001) or <8 weeks (SMD = −0.15, 95% CI: −0.39 to 0.09, *p* = 0.23). For sleep quality, no significant differences were shown between the interventions that lasted >15 days (57) (*p* = 0.33) and those that lasted ≤15 days (53) (*p* = 0.04). There was no significant difference in the effect of MBIs that lasted >15 days (SMD = −0.62, 95% CI: −1.88 to 0.64, *p* = 0.33) or those that lasted ≤15 days (SMD = −0.91, 95% CI: −1.80 to −0.02, *p* = 0.04).

### 3.6 Sensitivity analysis

In the sensitivity analysis (Table 3), we excluded the study by Krifa et al. (59) and observed a significant change in heterogeneity from 69% to 0%. Then, we excluded the study by Küchler et al. (51) and found that the level of heterogeneity also decreased to 0%, indicating a significant change. It is hypothesized that the outcome of stress indicators may have been the possible source of heterogeneity in both studies. Additional possible reasons include the inclusion of patients, specific treatment modalities, and inconsistencies in clinical indicators between domestic and foreign countries. The included patients and specific treatment modalities as well as the applied clinical indicators in these two literatures are as follows. Krifa et al. (59): Republic of Tunisia, aged 18–30 years, healthcare students, Internet-based positive psychology intervention, DASS-21. Küchler et al. (51): Germany, 18 years or older, undergraduate students, moderate to low mindfulness (FMI ≤ 37), PHQ-9, GAD-7, PSS-4, GAD-7, WHO. All of these different factors are possible sources of heterogeneity.

**Table 3:** Sensitivity analysis for stress symptom.

After excluding the reference	The Result of Heterogeneity:			
	Chi^2^	df	*p*	I^2^
Ahmad et al. (55)	19.30	5	0.002	74%
Anna F. Dawson et al. (58)	19.25	5	0.002	74%
Brian J. Hall et al. (57)	19.39	5	0.002	74%
Christo EI Morr et al. (52)	19.43	5	0.002	74%
Huberty et al. (56)	17.75	5	0.003	72%
Krifa et al. (59)	2.49	5	0.78	0%
Küchler, A.-M et al. (51)	2.68	5	*p* = 0.75	0%

### 3.7 Publication bias

We discovered that the funnel plots for depression, stress, anxiety, mindfulness scores and sleep quality were all symmetrical, indicating the absence of publication bias. Moreover, *p* > 0.05 indicate the absence of obvious publication bias. Begg’s tests (*p* = 0.02) and Egger’s regression (*p* = 0.037) also indicated a lack of publication bias.

## 4 Discussion

### 4.1 Discussion of pooled results

In this systematic review of studies including 1,824 participants, we found that MBIs significantly reduced depression (SMD=−0.33), anxiety (SMD=−0.35) and stress (SMD=−0.39) scores. Compared with the control interventions, MBIs had increased sleep quality and no significant effect on mindfulness scores in university students. Due to the statistical heterogeneity in the findings, the results should be interpreted with caution. This study explored each study’s effect on the overall risk via sensitivity analysis, thus investigating the main sources of heterogeneity. Major differences in intervention types, sample sizes (ranging from 52 to 386), intervention durations (ranging from 15days to 2months), intervention hours (ranging from 10 to 90min/week) on a weekly basis, type of control group (routine health care, waitlist, etc.), cultural background, measurement instruments, or other factors may contribute to heterogeneity. To our knowledge, this is the first meta-analysis and systematic review to evaluate the effectiveness of mindfulness therapy on five outcomes in university students. In our study, mindfulness therapy was found to significantly reduce depression, anxiety and stress symptoms, raise sleep quality, but it was not found to have a significant effect on mindfulness scores. Of course, mindfulness therapy does not have a magical effect on enhancing mindfulness rating scores and improving the sleep quality of college students.

### 4.2 Comparison of this study with other studies

To our knowledge, this is the first study to assess the association between MBIs and mental health (depression, anxiety, stress, mindfulness, and sleep quality) in university students. Previous studies have explored the relationship between college students with common mental health problems and mindfulness therapy. Huang et al. (63) performed a prospective study of mindfulness-based interventions and found that college students with depression and generalized anxiety disorder (GAD) showed improvements after receiving mindfulness-based interventions. Reangsing et al. (64) conducted a systematic review with university students and observed that online MBIs improved their depressive symptoms. Chen et al. (65) also reported that mobile mindfulness meditation (MMM) groups were more effective than control interventions at decreasing stress and alleviating anxiety. However, there was no difference in depression scores between the MMM and control groups. Previous studies have indicated inconsistencies in the effects of mindfulness therapy on depression symptoms. The current review included recent literature (2018–2023) and assessed five indicators of the effect of mindfulness therapy on mental health problems among college students. Previous studies have consistently shown positive effects of mindfulness therapy on stress symptoms. The current study also found an association between mindfulness and a lower likelihood of anxiety and depression Symptoms, and a higher likelihood of sleep quality(measured by the PSQI). However, there was no significant increase in mindfulness rating scores (measured by the GAD, MAAS, FFMQ, SF-12PCS and SF-12MCS).

### 4.3 The influence of MBIs on mood

Mindfulness originated from Buddhism, and the most frequently mentioned definition in the context of secular therapy is as follows: “awareness emerging through focusing, on purpose and nonjudgmentally, on the moment-by-moment experience unfolding” (66). Mindfulness-based cognitive therapy (MBCT) (67) and mindfulness-based stress reduction (MBSR) (11) are two prevailing programs based on mindfulness. Mindfulness is rooted in Eastern traditions. Due to the widespread and rapidly developing standardized MBI applications, mindfulness has recently become highly popular in Western psychology. To improve psychological functioning and well-being, the programs combine the essential practices of traditional mindfulness with the practices of contemporary psychology. A previous meta-analysis examined mindfulness as a mind–body exercise and found that the effects are primarily reflected in improved psychological well-being (9), individual emotion (68), mental health (9), symptoms of pain and depression and life quality (69). In addition, according to some studies, mindfulness therapy exerts positive influences on people with depression and anxiety (70) and affects the symptoms of anxiety among older adult individuals in their residential care (71), which is helpful for improving sleep quality (72). However, anxiety, depression (70), stress, and sleep quality (72) are very complex and multifactorial outcomes. In university students, anxiety, depression, stress, sleep quality and mindfulness are particularly varied and complex. As reported, common risk factors for mental disorders include female sex, senior age, nonreligious affiliation, unmarried/deceased parents, nonheterosexual behavior and identification, low ranking of secondary school, and college entrance extrinsic motivation among first-year college students (73). In addition, Becker et al. (74) reported that mental health symptoms are related to poor sleep. In contrast, current studies have pointed out that academic stress still plays an adverse role in mental health as well as the well-being of students (75). Accordingly, mental health is different among populations and is subject to different factors. According to our meta-analysis, mindfulness therapy had overall positive effects on reducing depression, anxiety, stress and increasing sleep quality; however, the benefit of improved mindfulness scores for university students was not significant due to many different factors. Therefore, mindfulness therapy needs to be further evaluated to determine its applicability among individuals experiencing mental health problems, including university students (61). Moreover, variance in the demographic characteristics of the participants across the 11 included studies may have contributed to the heterogeneity.

## 5 Limitations

This meta-analysis is the first to demonstrate that MBIs have a significant effect on reducing the depression, anxiety, and stress scores, and increasing the sleep quality scores of college students but have little effect on mindfulness scores.

In this study, there are several strengths. First, it was based on studies from 12 global databases. We only included RCTs, which made the samples more representative. Due to the large sample size, we performed a subgroup analysis and a sensitivity analysis. However, the limitations of this study should be considered. Due to the design of the systematic review, a causal relationship with a clear structure cannot be obtained. The outcome indicators of both pain (BPI score) and perceived social support (PSS score) also had an impact on the mental health of university students. However, with only one study reporting these outcomes, a comparison could not be made, so we could not include all outcome indicators. Third, although all included studies were randomized, the included studies’ blinding methods were seldom reported in detail. Because of the design limitations of the study, only four studies detailed the single blinding randomization method. Fourth, potential regulatory variables, such as intervention types, missing rates, and control group types, may impact the results to varying degrees. Because of the design limitations of the study, this study did not analyze the possible influencing factors in a stratified manner. Sixth, due to the number of studies included, the difficulty arising from carrying out more subgroup analyses may result in study heterogeneity. Fifth, at the academic level, this study was performed, but at the patient level, we find it is a difficult task to incorporate or address individual factors. Seventh, since the capability of detecting publication bias was limited by the quantity of included studies, it is impossible to rule out the possibility of publication bias. Eighth, for the same type of outcome index, no prevailing measurement instrument was available. Although SMD was selected as the indicator for effect size, attention should be given to the result during the interpretation.

### 5.1 Research and practical implications

For clinical practice, these findings have meaningful implications since the use of MBIs plays a role in depression, anxiety, stress and sleep quality among university students. First, further research is necessary on the methods of increasing participants’ motivation, reducing the missing rate, and maintaining the MBI effect. Second, due to the study limitations, it was impossible to perform subgroup analyses for the intervention types. Consequently, in-depth and stratified discussions and comparisons of different intervention types were conducted. In the coming years, it is necessary for researchers to conduct more quality studies with ample samples to verify the effectiveness of MBIs for menopausal women.

## 6 Conclusion

The results of this study indicated that mindfulness therapy may be associated with reduced depression, anxiety, and stress. Moreover, mindfulness therapy may increased the sleep quality of university students but did not significantly improve their mindfulness rating scores. The current findings emphasize the effects of mindfulness therapy on students’ moods. However, to verify the findings of the authors, further large-scale and prospective studies are necessary. In future studies, adherence and fidelity should be monitored to make the association exploration on a more extensive basis. There were trial heterogeneity and down-grading Jadad score, which led to the low evidence certainty. Therefore, it can only draw limited conclusions.

## Data availability statement

The original contributions presented in the study are included in the article/supplementary material, further inquiries can be directed to the corresponding author.

## Author contributions

ZXY: Conceptualization, Data curation, Formal analysis, Funding acquisition, Investigation, Methodology, Project administration, Resources, Software, Supervision, Validation, Visualization, Writing – original draft, Writing – review & editing. TY: Conceptualization, Data curation, Formal analysis, Funding acquisition, Investigation, Methodology, Project administration, Resources, Software, Supervision, Validation, Visualization, Writing – original draft, Writing – review & editing. CYF: Conceptualization, Data curation, Formal analysis, Funding acquisition, Investigation, Methodology, Project administration, Resources, Software, Validation, Visualization, Writing – original draft, Writing – review. ZZM: Data curation, Formal analysis, Methodology, Resources, Writing – original draft.
