# Supplementary figures and images for: The efficacy of mindfulness-based interventions on mental health among university students: a systematic review and meta-analysis
Source: Front Public Health. 2023 Nov 30;11:1259250. doi: 10.3389/fpubh.2023.1259250 (PMC10749636; doi:10.3389/fpubh.2023.1259250)

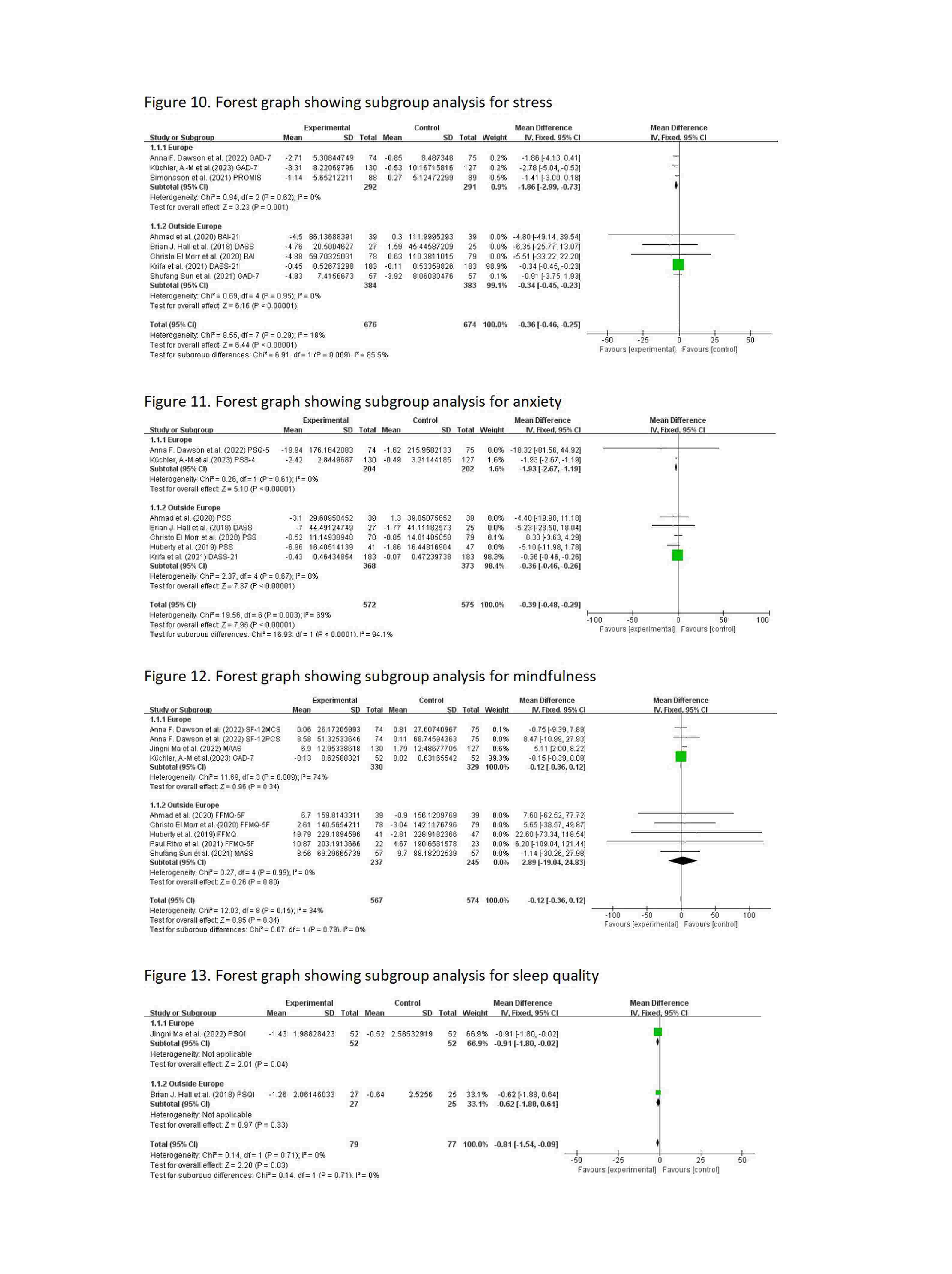

Supplement: Supplementary file 1 [file Image_1.jpg]

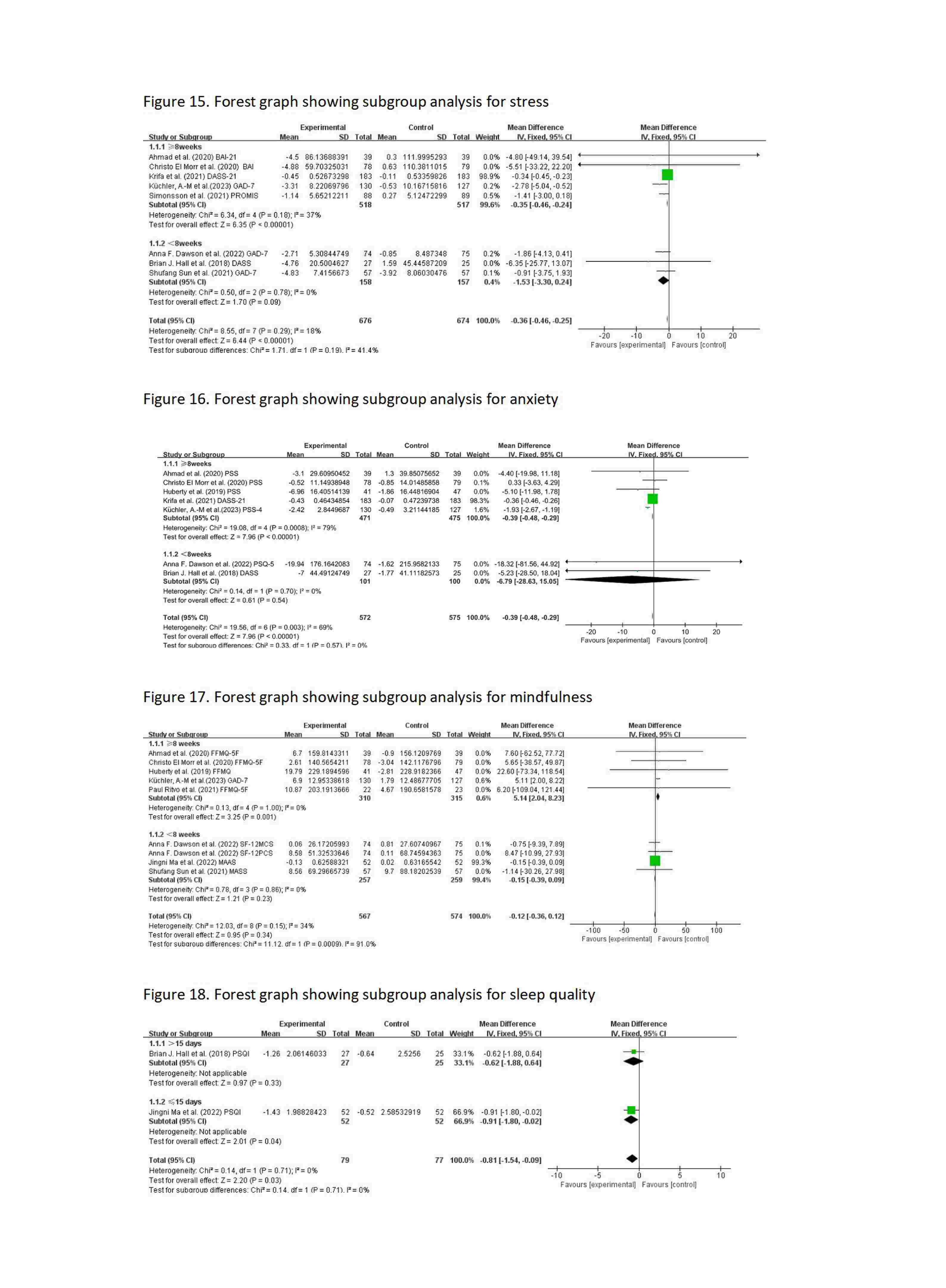

Supplement: Supplementary file 2 [file Image_2.jpg]

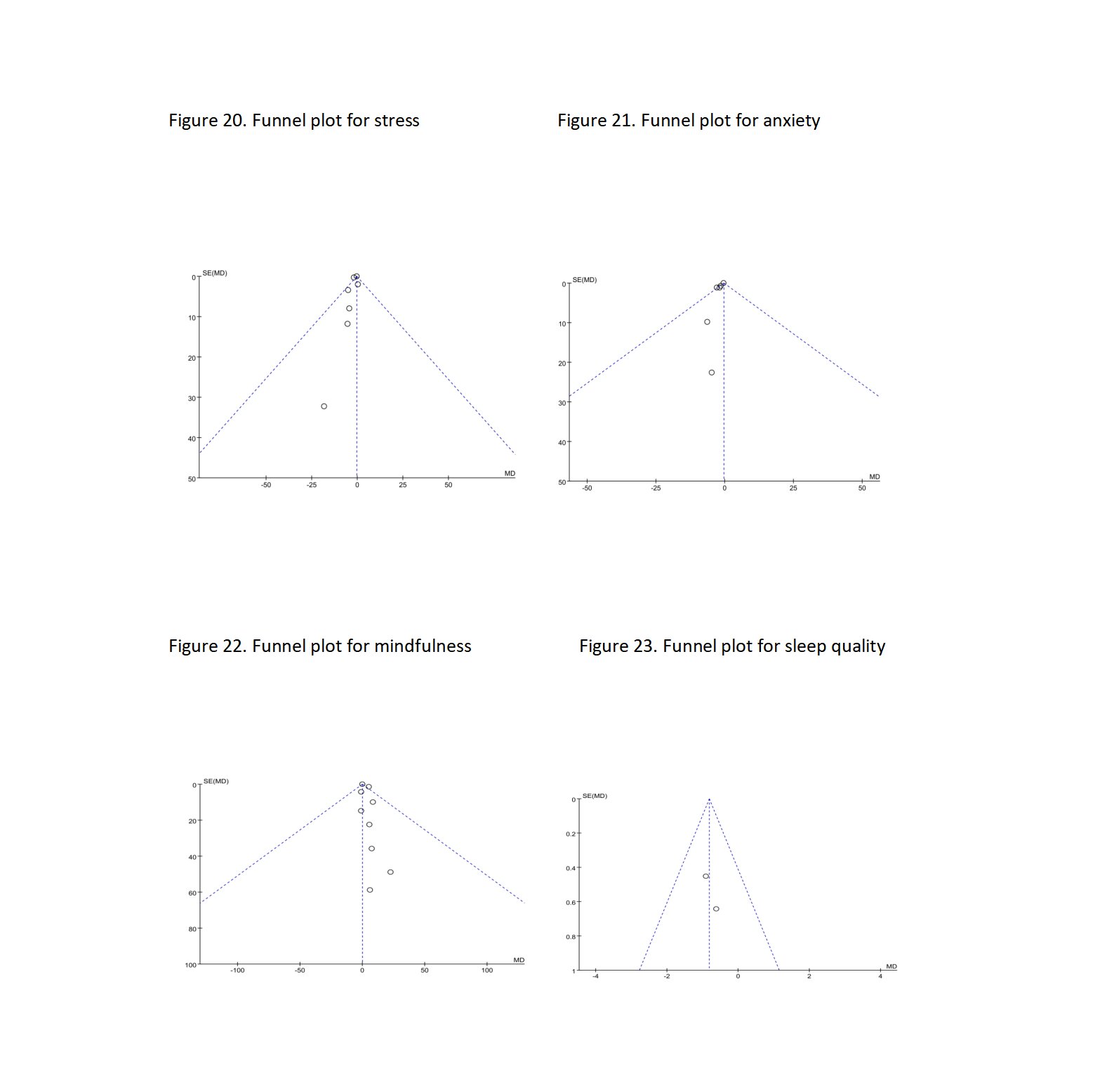

Supplement: Supplementary file 3 [file Image_3.jpg]
